# 维吾尔族肺腺癌患者的*EGFR*基因突变分析

**DOI:** 10.3779/j.issn.1009-3419.2013.02.04

**Published:** 2013-02-20

**Authors:** 莉 单, 琰 张, 峰 赵, 立谋 郑, 国庆 张

**Affiliations:** 1 830000 乌鲁木齐，新疆医科大学附属肿瘤医院 Affiliated Cancer Hospital, Xinjiang Medical University, Urumuqi 830000, China; 2 361005 厦门，厦门大学药学院 School of Pharmaceutical Sciences, Xiamen University, Xiamen 361005, China

**Keywords:** 肺肿瘤, EGFR, 实时荧光PCR, 基因突变, Lung neoplasms, Epidermal growth factor receptor, Real-time polymerase chain reaction, Gene mutation

## Abstract

**背景与目的:**

表皮生长因子受体（epidermal growth factor receptor, EGFR）是一种跨细胞膜糖蛋白，属于受体型酪氨酸激酶家族。以吉非替尼为代表的EGFR酪氨酸激酶抑制剂对于*EGFR*突变的肺癌患者显示出良好的治疗效果，然而*EGFR*的突变率在不同民族和不同种族的人群中表现出较大差异。本研究旨在分析维吾尔族中肺腺癌患者肿瘤组织的*EGFR*基因突变情况，同时比较维吾尔族与汉族肺腺癌患者肿瘤组织*EGFR*基因突变率的差异性。

**方法:**

收集临床肺腺癌患者石蜡包埋组织标本138例，包括68例维吾尔族和70例汉族肺腺癌的样本，采用ARMS（amplification refractory mutation system, ARMS）PCR扩增方法检测*EGFR*基因外显子18、19、20及21的突变，χ^2^分析对比维吾尔族和汉族肺腺癌*EGFR*基因突变差异。

**结果:**

138例肺腺癌患者中有43例*EGFR*基因突变，总突变率为31.2%，其中维吾尔族突变11例，突变率为16.2%，汉族突变32例，突变率为45.7%，维吾尔族肺腺癌*EGFR*突变率与汉族肺腺癌*EGFR*突变率比较有明显差异（*P* < 0.001），突变以外显子19-del和L858R为主要突变点。

**结论:**

维吾尔族中肺腺癌*EGFR*基因突变率为16.2%，汉族中*EGFR*基因突变率为45.7%，维吾尔族肺腺癌*EGFR*突变率明显低于我国汉族*EGFR*基因突变。

## Abstract (en)

**Background and objective:**

Epidermal growth factor receptor (EGFR), a transmembrane protein, is a member of the tyrosine kinase family. Gefitinib, an EGFR tyrosine-kinase inhibitors, has shown a high response rate in the treatment of lung cancer in patients with *EGFR* mutation. However, significant differences in *EGFR* mutations exist among different ethnic groups. The aim of this study is to investigate the prevalence of *EGFR* mutations in Uighur lung adenocarcinoma patients by using a rapid and sensitive detection method and to analyze *EGFR* mutation differences compared with Han lung adenocarcinoma patients.

**Methods:**

We examined lung adenocarcinoma tissues from 138 patients, including 68 Uighur lung adenocarcinoma patients and 70 Han lung adenocarcinoma patients, for *EGFR* mutations in exons 18, 19, 20, and 21 by using the amplification refractory mutation system (ARMS) PCR method. The mutation differences between Uighur and Han lung adenocarcinoma were compared by using the *chi-square* test method.

**Results:**

*EGFR* mutations were detected in 43 (31.2%) of the 138 lung adenocarcinoma patients. *EGFR* mutations were detected in 11 (16.2%) of the 68 Uighur lung adenocarcinoma patients and in 32 (45.7%) of the 70 Han lung adenocarcinoma patients. Significant differences were observed in the *EGFR* mutations between Uighur lung adenocarcinoma patients and Han lung adenocarcinoma patients (*P* < 0.001).

**Conclusion:**

Our results indicate that the *EGFR* mutation in Uighur lung adenocarcinoma patients (16.2%) is significantly lower than that in Han lung adenocarcinoma patients (45.7%).

肺癌是世界范围内常见的癌症，发病率和死亡率居各癌症之首。据2010年我国卫生部的统计，肺癌死亡率为30.83/10万。肺癌已经成为发病率和死亡率第1位的恶性肿瘤，其5年生存率仅为15%^[1-3]^。表皮生长因子（epidermal growth factor receptor, EGFR）是一种跨细胞膜糖蛋白，属于受体型酪氨酸激酶，EGFR信号通路在细胞的生长、增殖和分化等生理过程中发挥着重要调节作用。*EGFR*基因在多种肿瘤中存在突变或过表达，并通过其介导的信号通路调控肿瘤的生长、侵袭、转移和血管新生^[4-6]^。以吉非替尼为代表的EGFR酪氨酸激酶抑制剂（tyrosine kinase inhibitors, TKIs）靶向药物在肺癌的治疗中显示出良好的治疗效果，然而，该类药物的治疗效果在不同个体间存在很大差异。多项临床研究^[5, 7-12]^显示，*EGFR*基因外显子18-21突变是导致不同个体间治疗效果差异的主要原因。因此，根据*EGFR*基因突变检测结果选择合适的治疗对象，对于提高患者治疗效果、延长患者的生存期有重要意义。目前，已有的肺癌*EGFR*突变研究还没有针对维吾尔族突变率的报道。本研究检测了68例维吾尔族和70例汉族肺癌患者的*EGFR*基因，首次报道了维吾尔族肺癌*EGFR*基因突变率，并对维吾尔族和汉族*EGFR*基因突变率进行了比较。

## 1 材料与方法

### 1.1 研究对象

收集医院维吾尔族肺腺癌患者石蜡包埋组织标本68例，其中男性39例，女性29例；年龄30岁-75岁，中位年龄52岁。汉族肺腺癌患者石蜡包埋组织标本70例，其中男性49例，女性21例；年龄35岁-79岁，中位年龄57岁。

### 1.2 标本采集与DNA提取

采用病理石蜡切片样品，5 μm切片，不少于8片，室温保存。所用样品均为病理诊断确定含有肿瘤组织的蜡块切片，并且保存时间不超过两年。取石蜡标本组织，用QIAGEN公司核酸提取试剂盒提取（QIAamp® DNA FFPE Tissue Kit，商品号：564074）DNA，所提DNA溶于Tris-HCl（10 mmol/L, pH8.0），经紫外分光光度计检测提取质量，并确定浓度，用Tris-HCl溶液（10 mmol/L, pH8.0）调整DNA浓度到10 ng/µL和2 ng/µL备用。

### 1.3 实时荧光PCR扩增

采用ARMS（amplification refractory mutation system, ARMS）方法进行PCR扩增，检测*EGFR*基因外显子18、19、20和21突变。用厦门艾德生物医药科技公司*EGFR*基因突变检测试剂盒（人类*EGFR*基因21种突变检测试剂盒）进行检测，实验具体操作步骤参照试剂盒说明书进行操作。采用StrataGene MX3000P实时PCR仪进行扩增，每次检测样品包括1个阳性质控品、1个NTC对照。如果Ct值为0或Ct值＞30，则实验结果判为野生型。所述荧光PCR的反应条件：95 ℃预变性5 min，1个循环；95 ℃变性25 s，64 ℃退火20 s，72 ℃延伸20 s，15个循环；93 ℃变性25 s，60 ℃退火35 s，72 ℃延伸20 s，31个循环。

### 1.4 统计学分析

采用SPSS 13.0统计软件进行统计学分析，采用χ^2^和*Fisher*精确检验分析突变差异，*P*＜0.05为差异有统计学意义。

## 2 结果

共检测138例肺腺癌的样本，包括68例维吾尔族、70例汉族，共检测出43例突变，检测总突变率为31.2%。维吾尔族突变11例，突变率为16.2%，汉族突变32例，突变率为45.7%，以外显子19和21突变较为多见。维吾尔族*EGFR*突变率明显低于我国汉族*EGFR*突变率（χ^2^=14.03, *P*＜0.001），结果见表 1，突变比例分析图见图 1。

**1:** 维吾尔族与汉族肺腺癌*EGFR*基因突变统计结果 *EGFR* mutation comparison between Uighur lung adenocarcinoma and Han lung adenocarcinoma

*EGFR* gene	Uighur lung adenocarcinoma	Han lung adenocarcinoma	*P*
*EGFR* mutations	11	32	
EGFR wild-type	57	38	< 0.001
Total	68	70	

**1:**
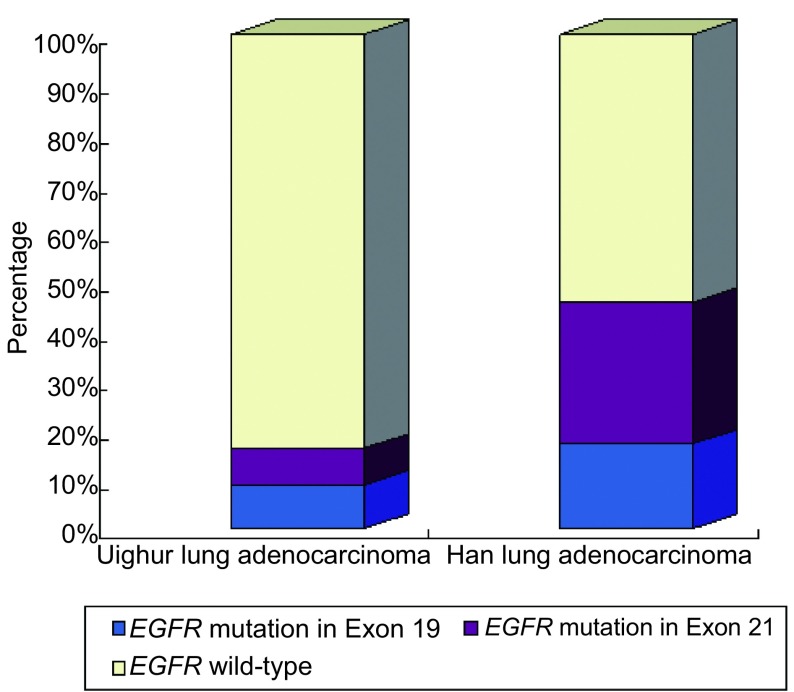
维吾尔族与汉族肺腺癌*EGFR*基因突变比较 Histogram comparison of *EGFR* mutations between Uighur lung adenocarcinoma and Han lung adenocarcinoma. EGFR: epidermal growth factor receptor.

在检测的肺腺癌*EGFR*基因突变中，突变点包括19-del突变和外显子21的*L858R*突变，其中维吾尔族外显子19-del突变6例，占突变数的54.5%，*L858R*突变5例，占突变数的45.5%。汉族19-del突变12例，占突变数的37.5%。*L858R*突变20例，占突变数的62.5%。维吾尔族外显子19和外显子21（L858R）的突变率与汉族外显子19和外显子21（L858R）的突变率相比无明显差异（*P*=0.48）（表 2）。

**2:** *EGFR*基因外显子19与外显子21突变分析 The analysis of *EGFR* mutations in Exon 19 and Exon 21

*Mutated* EGFR	Uighur lung adenocarcinoma	Han lung adenocarcinoma	*P*
Exon 19-deletion	6	12	
Exon 21-L858R	5	20	0.48
Total	11	32	

## 3 讨论

EGFR属于表皮生长因子ErbB家族，该家族包括HER1、HER2、HER3和HER4四个成员。EGFR是一种细胞表面膜蛋白受体，由1, 186个氨基酸组成，分子量为170 kDa，由三个功能不同的结构域组成，包括胞外区、跨膜区和细胞内的酪氨酸区^[13]^。当EGFR与其相应的信号分子结合后，受体分子之间会形成二聚体，两个受体胞内的酪氨酸激酶彼此作用而相互磷酸化，并启动胞内的级联信号通路，从而引起细胞内一系列形态学的改变^[13-15]^。

*EGFR*基因在多种肿瘤会存在突变或者过表达，并通过其下游信号通路调控肿瘤的生长、增殖和转移。目前，常用EGFR-TKIs主要包括吉非替尼和厄洛替尼，是治疗肺癌最有效的靶向药物。然而，该类药物对*EGFR*突变患者的有效率高达80%以上，而对野生型患者几乎没有疗效，因此，检测*EGFR*的突变情况是肺癌化疗预后重要标志物。研究表明，不同人种肺癌的*EGFR*突变率差异较大，Schmid等^[16]^和Kris等^[17]^通过对大量肺癌病例研究发现，高加索人（Caucasian）肺癌*EGFR*突变率约为7%-17%。Li等^[18]^通过对202例肺腺癌患者发现，我国汉族人的肺腺癌*EGFR*突变率为75.3%。Han^[19]^和Hsu等^[20]^的研究进一步表明，东亚人的肺腺癌*EGFR*突变率约为30%-62%。本研究首次对维吾尔族肺腺癌*EGFR*突变进行检测，检测结果表明维吾尔族肺腺癌*EGFR*突变率为16.2%，其突变率明显低于我国汉族肺腺癌*EGFR*突变率，略高于高加索人*EGFR*突变率。本研究对维吾尔族肺腺癌*EGFR*突变检测为临床医生提供了重要参考信息，有益于其肺腺癌的个体化治疗。

从地域分布来看，汉族人与日本人居源于东亚大陆，高加索人居源于欧洲大陆，维吾尔族人居于欧洲大陆和东亚大陆之间。居源于东亚大陆人肺腺癌*EGFR*突变率较高，居源于欧洲大陆的人肺腺癌*EGFR*突变率较低，维吾尔族人所居区域突变率介于两者之间，肺腺癌*EGFR*突变率从欧洲大陆到东亚大陆表现出突变率逐渐增大的趋势。

肺腺癌*EGFR*基因外显子19缺失突变是吉非替尼类药物作用的关键靶点，其缺失突变可引起酪氨酸激酶结构域中4个氨基酸残基的缺失，从而导致EGFR-ATP受体结合域的角度改变，使吉非替尼类药物更易结合到该位点，通过阻止ATP进入而阻断EGFR激酶活性^[21]^。本研究检测了68例维吾尔族和70例汉族肺腺癌*EGFR*基因，发现*EGFR*总突变率为31.2%，维吾尔族的突变率为16.2%，其中外显子19-del突变的突变率较高，占突变比例的54.5%。在汉族中*EGFR*突变率为45.7%，其中外显子19的缺失突变的突变率有12例，占突变比例的37.5%，维吾尔族*EGFR*基因外显子19缺失突变的突变率略高于汉族的外显子19缺失突变的突变率，分析表明维吾尔族肺腺癌*EGFR*基因外显子19突变率与汉族外显子19突变率差异无统计学意义（*P*=0.48）。

综上所述，本研究表明维吾尔族肺腺癌患者*EGFR*基因突变率明显低于我国汉族*EGFR*基因突变率。*EGFR*基因突变与EGFR-TKIs药物治疗敏感性明显相关，基于*EGFR*的突变情况来选择EGFR-TKIs的药物进行化疗已成为临床医生的共识，本研究首次提供维吾尔族肺腺癌的*EGFR*突变率信息，可以为临床医生提供有益参考，使广大肺腺癌患者受益。
